# Characteristics and Fitness Analysis through Interspecific Hybrid Progenies of Transgenic *Brassica napus* and *B. rapa* L. ssp.

**DOI:** 10.3390/ijms231810512

**Published:** 2022-09-10

**Authors:** Soo-In Sohn, Senthil Kumar Thamilarasan, Subramani Pandian, Young-Ju Oh, Hyeon-Jung Kang, Eun-Kyoung Shin

**Affiliations:** 1 Department of Agricultural Biotechnology, National Institute of Agricultural Sciences, Rural Development Administration, Jeonju 54874, Korea; 2 Institute for Future Environment Ecology Co., Ltd., Jeonju 54883, Korea

**Keywords:** *Brassica napus*, *Brassica rapa*, genetically modified crops, interspecific hybridization, transgene persistence, SSR markers

## Abstract

Interspecific hybridization between transgenic crops and their wild relatives is a major concern for transgene dispersal in the environment. Under controlled conditions, artificial hand pollination experiments were performed in order to assess the hybridization potential and the fitness of interspecific hybrids between *Brassica rapa* and genetically modified (GM) *Brassica napus*. Initially, six subspecies of *B. rapa* were hybridized with GM *B. napus* through hand pollination. In the resulting F_1_ hybrids, the combination of *B. rapa* ssp. *narinosa* (♀) × GM *B. napus* (♂) had the highest crossability index (16.9 ± 2.6). However, the F_1_ selfing progenies of *B. rapa* ssp. *rapa* (♀) × GM *B. napus* were found to be more effective in producing viable future generations with the highest crossability index (1.6 ± 0.69) compared to other subspecies. Consequently, they were used for the generation of F_2_ and F_3_ progenies. The 18 different morphological characteristics among the parental cross-combinations and F_1_ hybrid progenies were measured and visualized through hierarchical clustering. Different generations were found to be grouped based on their different morphological characteristics. The chromosome numbers among the interspecific hybrids ranged from 2n = 29 to 2n = 40. Furthermore, the SSR markers revealed the presence of genomic portions in the hybrids in comparison with their parental lines. There is a high possibility of transgene flow between GM *B. napus* and *B. rapa*. The study concluded that the interspecific hybrids between *B. napus* and *B. rapa* can be viable and can actively hybridize up to F_3_ generations and more. This suggests that the GM *B. napus* can disperse the transgene into *B. rapa*, and that it can pass through for several generations by hand pollination in a greenhouse environment.

## 1. Introduction

The commercialization of genetically modified (GM) crops started in 1996. The global cultivation area of GM crops has increased dramatically in the last 25 years. The production has also increased dramatically in the last 25 years. The production has experienced over a 100-fold increase [1,2]. However, the main problem is potential transgene flow from GM crops that can affect non-transgenic counterparts, such as closely related or sexually compatible species [3]. Thus, the concerns about the gene flow from GM crops to their wild relatives have been intensified in the countries where their commercial cultivation is authorized. The *Brassicaceae* family is getting special attention because it has wild relatives throughout the world, and it can hybridize with any close and distant relatives within genera and species [4,5]. In the *Brassicaceae*, there are six *Brassica* species with three different genomes (A (n = 10); B (n = 8); C (n = 9)), which include three diploid species, namely *Brassica rapa* (AA, 2n = 20), *B. nigra* (BB, 2n = 16), and *B. oleracea* (CC, 2n = 18), through a natural hybridization process which further formed three allotetraploid species, namely *B. juncea* (AABB, 2n = 36), *B. carinata* (BBCC, 2n = 34), and *B. napus* (AACC, 2n = 38) [6]. Among the crops, *B. napus* (oilseed rape) is one of the most preferred and suitable for gene flow studies, since it can produce a large amount of pollen and has a huge number of related species, including cultivars and wild relatives [7]. Several studies have shown sexually compatible relatives with this crop: *B. rapa* [8,9], *B. juncea* [10], *B. oleracea* [11], *Hirschfeldia incana* [12,13], *Sinapis arvensis* [12,14], and *Raphanus raphanistrum* [13,15] have been reported. Most of the commercial GM *B. napus* have potential transgenes that are resistant to herbicides such as glyphosate, glufosinate, and bromoxynil [16]. Selective pressure on herbicides promotes the growth of GM *B. napus* and increases the risk of the escape of herbicide resistance genes through hybridization with related species [17]. The probability of establishing a transgene with another species depends largely on the suitability of the F_1_ hybrid between the crop and wild species and subsequent generations. Despite the classical view that wild crop hybrids should be less suitable than their parents, there are instances when wild crop hybrids may be as suitable or better suited as their parents [18,19,20,21,22].

The majority of gene flow studies on GM *Brassica* sp. have focused on crosses between transgenic *B. napus* (2n = 38; AACC) and wild relative *B. rapa* (2n = 20; AA) [5,23,24]. Spontaneous hybridization occurs in Europe and the United States, and their generations can easily backcross to *B. rapa* in wild environments [3,25,26]. However, limited information is known about the consequences of invasion between *B. napus* and *B. rapa*, and gene establishment is not well documented [27]. Cross-compatibility and callose deposition in pollen tubes are the main reasons for hybridization failure in Brassica [28]. However, reports of artificial hand pollination which has resulted in crop and relative hybridization are important sources of knowledge because they enable the evaluation of species’ reproductive compatibility and the identification of hostile species combinations. This makes it easier for us to perform a cautious examination of the species that ought to be taken into account for their potential to serve as transgene escape targets in the local environment [5,29,30]. Most of the studies to evaluate the gene transfer from *B. napus* to *B. rapa* were conducted in the F_1_ and BC_1_ generations. Moreover, few studies have been conducted to investigate the fate of transgenes for more than three generations of interspecific hybridization. To assess whether a transgene can increase persistence across all generations through interspecific hybridization, the frequency of hybridization between the two related species, which increased their fitness, survival rates, and fertility, should be considered in subsequent generations [6,29,31].

Since Korea is one of the prime exporters of diversified *B. rapa* ssp., the possibility of transgene flow and ecological sustainability from GM rapeseed to *B. rapa* should be investigated. Therefore, in this study, we tried to analyze the possibility of gene transfer between GM rapeseed and various subspecies of *B. rapa*, and the diversity of subsequent generations and reciprocal combinations of interspecific hybrids. To this end, the main objectives of the study are: (i) the assessment of crossability indices between GM *B. napus* and six subspecies of *B. rapa* through artificial hand pollination; (ii) the morphological characteristics revealing their relative fitness characters for transgene persistence in generational progress; (iii) chromosome counts of individuals of F_1_, F_2_, F_3_ progenies; and (iv) the inspection of the genetic similarity using SSR markers for F_1_, F_2_, F_3_, and BC_1_ progenies.

## 2. Results

### 2.1. Cross-Compatibility of Each B. rapa ssp. with Transgenic B. napus

Three crossing experiments were performed based on the flowering times of three different sets of GM *B. napus* with *B. rapa* ssp. The artificial hand pollination of six subspecies resulted in an average of 1101 flowers, leading to a 45.3% pod-setting ratio, and an average of seven seeds were obtained from each pod. In parental lines, the maximum crossability index was observed in *B. rapa* ssp. *chinensis* (25.1 ± 2.3) (Table 1). Despite this, the maximum crossability index of 16.9 ± 2.6 was observed during initial hybridization in *B. rapa* ssp. *narinosa* (♀) × GM *B. napus* (♂) parental cross-combinations (Table 1). That produced an average number of seeds in each pod with a 58.8% pod-setting ratio. However, there was no significant difference in producing further generations among the cross-combinations of all the subspecies with GM *B. napus*. Among them, the F_1_ hybrid (selfing) of *B. rapa* ssp. *rapa* had the highest crossability index (1.6 ± 0.7) compared to other subspecies (Table 1). Therefore, *B. rapa* ssp. *rapa* was taken into F_2_ hybrid and F_3_ hybrid (selfing) generations, and the resulting crossability indexes were 2 ± 0.7 and 6.4 ± 4.8, respectively (Table 1).

The hybridization of reciprocal combinations resulted in comparatively higher crossability index values, among which, the maximum crossability index was found between GM *B. napus* (♀) × *B. rapa* ssp. *nipposinica* (♂) (27.5 ± 2.9), with a ratio of 17 seeds per pod. Inclusively, the statistical analysis with one-way ANOVA shows that the crossability index was highly significant with respective crossing materials (*p* < 0.05). A multiple comparison with the Tukey test reveals that differences in the average crossability indexes were largely attributable to parental, cross-combination, and F_1_ hybrids (Table 1). The occurrence of vivipary in parental combinations of *B. rapa* ssp. (♀) was found to be at higher rates, ranging from 22.7% to 73%. In contrast, reciprocal combinations of GM *B. napus* (♀) showed less vivipary, and ranged from 0.1% to 4.2% (Table 1 and Supplementary Table S1).

### 2.2. Morphological Characteristics and Relative Fitness of Parental Genotypes and Interspecific Hybrids

**Figure 1:**
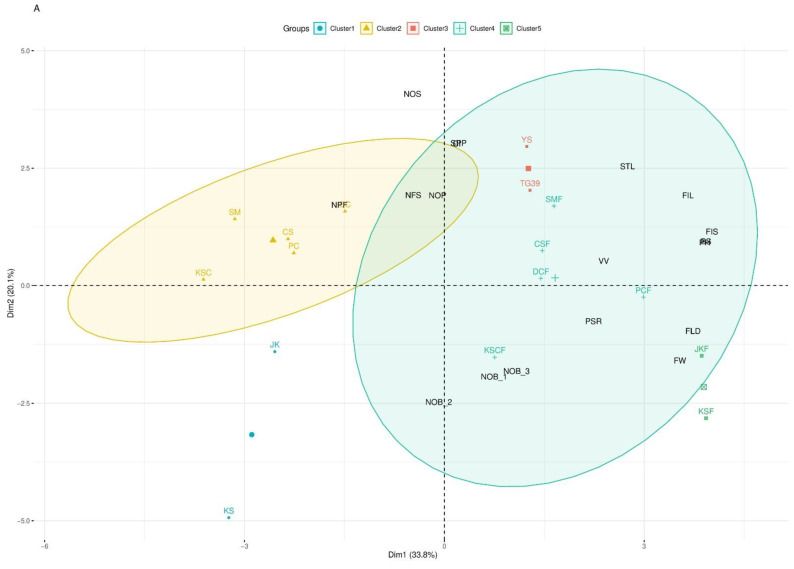

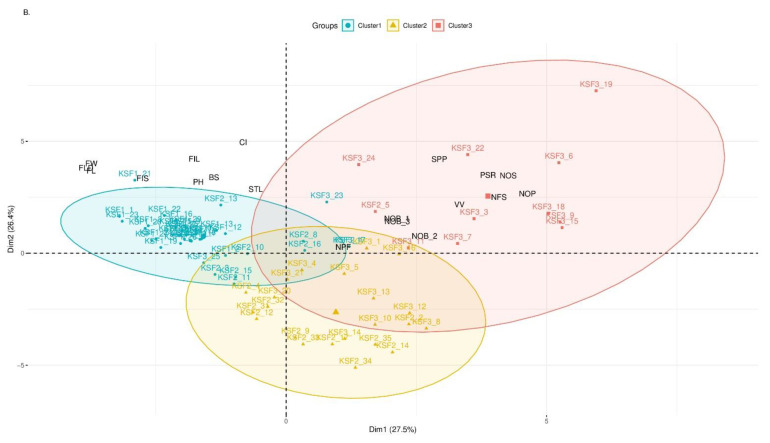
Morphological characteristics represented as variables in these clusters. (**A**) Representation of parental and cross-combination of *B. rapa* (♀) × GM *B. napus* (♂) (PCC). (**B**)**.** Representation of *B. rapa* ssp. *rapa* (♀) × GM *B. napus* (♂) (KSF_1_ to KSF_3_).

Based on 18 morphological characteristics, the parental and all the crossing materials were grouped into two data sets. The *B. rapa* ssp. and their respective cross-combinations with GM *B. napus* (parental cross-combinations/PCC) are included in one group, whereas another one with F_1_, F_2_, and F_3_ selfing progenies of *B. rapa* ssp. *rapa* (♀) × GM *B. napus* (♂) (interspecific hybrids) is included in another group. The hierarchical clustering of parental cross-combinations (Figure 1A) revealed the presence of five clusters. As expected, the parents, *B. rapa* ssp. *Pekinensis* and *B. rapa* ssp. *Rapa*, formed cluster 1. Cluster 2 encompassed a second set of parents (*B. rapa* ssp. *Nipposinica*, *B. rapa* ssp. *Oleifera*, *B. rapa* ssp. *Parachinensis*, *B. rapa* ssp. *Chinensis*, and *B. rapa* ssp. *Narinosa*) marked by high values of generative characters such as NPF (no. of pollinated flowers). Cluster 3 was grouped with GM *B. napus* (TG#39) and non-GM *B. napus*, which exhibited the no. of seeds (NOS) and the no. of second branches (NOB_2). Cluster 4 clearly distinguishes cross-combinations of (*B. rapa* ssp. (♀) × GM *B. napus* (♂)) genotypes, exhibiting strong vivipary (VV), long style (STL), number of branches (NOB_1), and filament (FL) characteristics. Finally, cluster 5 highlighted the cross-combinations *B. rapa* ssp. *pekinensis* (♀) × GM *B. napus* (♂) and *B. rapa* ssp. *rapa* (♀) × GM *B. napus* (♂), harboring longer and wider flowers (FW, FL, and FD) (Supplementary Table S2).

Regarding the selfing progenies of *B. rapa* ssp. *rapa* (♀) × GM *B. napus* (♂) (KSF_1_ to KSF_3_) interspecific hybrid classification, a total of three clusters have been inferred (Figure 1B). Cluster 1 (40 progenies) indicated the individuals presenting long and wide flowers, whereas cluster 2 (22 progenies) informed about the plant architecture regarding pod-setting ratio, branches, and plant height. Cluster 3 (11 progenies) grouped individuals showing better reproductive fitness, with higher values of the number of seeds, number of pods, and pod-setting ratio (Supplementary Figure S1 and Table S3). Overall, good reproductive fitness was observed for the cluster 3 of parental cross-combinations (PCC) and the interspecific hybrids (Figure 1A,B), suggesting a good fitness of generative agricultural characteristics that can help us assess the further generations.

### 2.3. Chromosome Numbers of Interspecific Hybrids and Progenies

**Figure 2:**
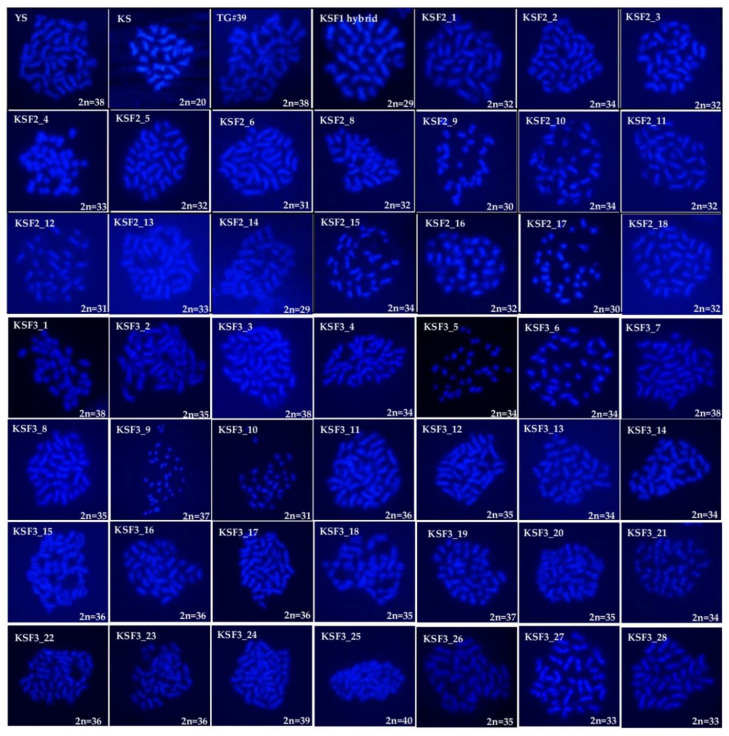
Chromosome counts on non-GM *B. napus* (YS), *B. rapa* ssp. *rapa* (KS), and GM *B. napus* (TG#39). Cross-combination of *B. rapa* ssp. *rapa* (♀) × GM *B. napus* (♂) F_1_ hybrids and selfing generation of KSF_2_ and KSF_3_.

The parental genotypes, *B. rapa* ssp. *rapa* (KS) KSF_1_ hybrid and the selfing progenies of KSF_2_ and KSF_3_, were found to have variable chromosome numbers in microscopic observation (Figure 2). The chromosome numbers of parental genotypes, such as *B. rapa* ssp. *rapa* (KS) (2n = 20), GM *B. napus* (2n = 38), and non-GM *B. napus* Youngsan (YS) (2n = 38), are used as internal control. The KSF_1_ hybrid revealed that 2n = 29 as (AAC; n+n), which were derived from the hybridization of *B. rapa* ssp. *rapa* (n = 10) and GM *B. napus* (n = 19). The respective KSF_1_ hybrids of AAC parents were used to generate further KSF_2_ (selfing of KSF_1_) progenies that showed a range of chromosome numbers from 2n = 29 to 2n = 34. In KSF_3_ (selfing of KSF_2_) progenies, the chromosome numbers varied from 2n = 31 to 2n = 40. For the KSF_2_ and KSF_3_ selfing progenies, the plants showed a particular chromosome number in large proportions, having 2n = 32 (7), 2n = 34 (6), and 2n = 36 (6), respectively. The most predominant chromosome numbers of the selective selfing progenies on KSF_2_ and KSF_3_ are 31 and 35, respectively. However, in KSF_3_, three progenies have occurred with 2n = 38 chromosome numbers, which has increased over successive generations.

### 2.4. Assessment of Intergenomic Recombination and Their Progenies Validation by Using SSR Markers

**Figure 3:**
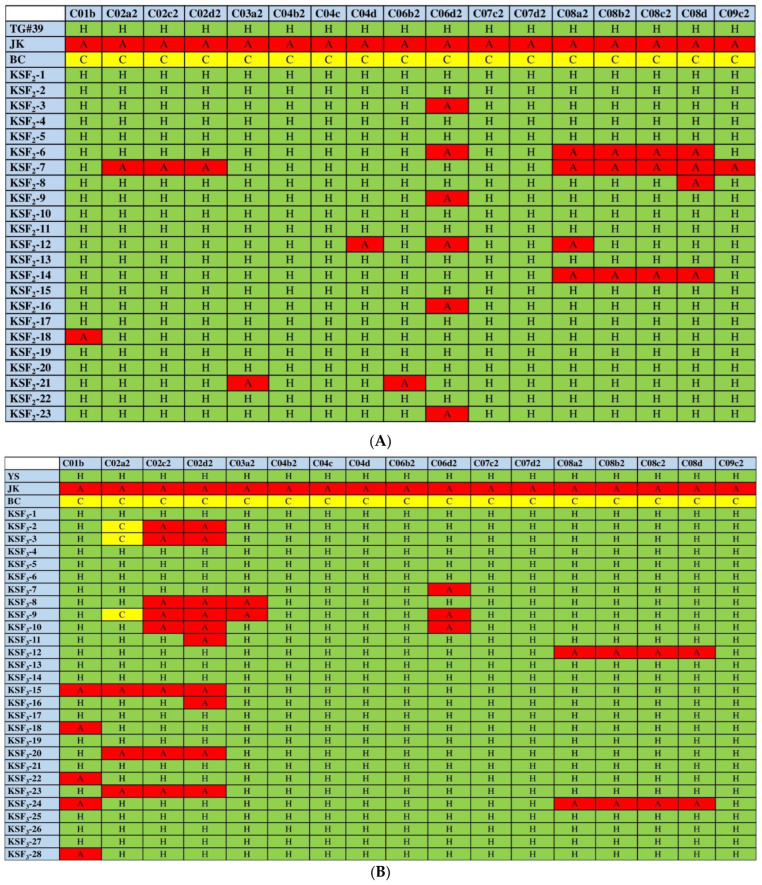
Chromosome segregation analysis by chromosome-specific SSR markers, which are indicated by bold alphanumeric characters in the first horizontal row. (**A**). F_2_ generation of *B. rapa* ssp. *rapa* (♀) × GM *B. napus* (♂) (KSF). (**B**). F_3_ generation of *B. rapa* ssp. *rapa* (♀) × GM *B. napus* (♂) (KSF). A, C, and H indicate the A chromosome, C chromosomes (highlighted in yellow), and hybrid-type bands, respectively. The C chromosome was identified and highlighted in green; the intergenomic recombination-induced loss of C chromosomal regions was identified and highlighted in red.

To validate the interspecific hybrids and their selfing progenies with the above-mentioned morphological characteristics and chromosome number variations, the 17 SSR markers were used for the genetic analysis of 23 KSF_2_ and 28 KSF_3_ selfing progenies and 33 KSBC_1_ plants. KSF_2_ and KSF_3_ extensively revealed a heterozygous nature. As shown in Figure 3A,B, 93.09% and 90.23% of KSF_2_ and KSF_3_ were found to be of a heterozygous nature (presence of the marker in both A and C genomes). In the A and C genomes of the KSF_2_ and KSF_3_ plants, fewer SSR loci were missed (Figure 3A,B). Contrarily, in KSBC_1_ plants, only 53.65% were found to be heterozygous. Due to homeologous recombination, the A genome (46%) was found to be in higher frequencies in KSBC_1_ than in KSF_2_ and KSF_3_ hybrid progenies (Supplementary Figure S2).

Cluster analysis using the Jaccard distance matrix was used to evaluate the SSR marker data. The maximum distance was found in backcross generations (0.971), and a minimum distance (0.029) was recorded on KSBC_1_, KSF_2_, and KSF_3_ generations. Using the distance matrix, the UPGMA dendrogram was constructed, which revealed a good degree of fit by the values of the cophenetic correlation coefficient (r = 0.940, *p* < 0.001) (Supplementary Figure S3). The KSF_2_ and KSF_3_ hybrids and KSBC_1_ progenies were clustered into eight major clusters. This is in accordance with the tree constructed with 18 morphological traits (Supplementary Figure S1). The parental plants of *B. rapa* ssp. *rapa* were clustered with KSBC_1_ progenies, whereas *B. napus* and KSF_1_ were grouped with KSF_2_ progenies. Similarly, the control plants of *B. oleracea* were clustered separately and out-branched far from all other clusters.

## 3. Discussion

Many studies have explored the interspecific hybridization and gene flow between transgenic *B. napus* and various subspecies and varieties of *B. rapa* [32,33,34]. In our previous report, the gene flow of an early flowering gene (*BrAGL20*) was characterized in F_1_ hybrids between *B. rapa* ssp. *pekinensis* and GM *B. napus* [29]. Apart from F_1_ hybrids, there are no reports on selfing progenies’ transgene persistence in subsequent generations (F_2_ and F_3_). It is critical to investigate transgene persistence over multiple generations. Hence, in this study, to reveal the gene flow of the transgene to more generations, interspecific hybridization of six *B. rapa* ssp. and GM *B. napus* (as a paternal) was performed through artificial hand pollination. Several subspecies of *B. rapa* are known for their higher levels of phenotypic and genetic diversity. They can have varying degrees of cross-compatibility and self-incompatibility by nature [5,35,36]. However, we preliminarily investigated the fertilization barriers or self-incompatibilities that occurred during self-pollination in six subspecies. However, through artificial hand pollination, they showed no self-incompatibility with the flower buds. Different levels of crossability have been recorded for each subspecies (Table 1). In cross-combination, the average crossability of *B. rapa* ssp. with GM *B. napus* is four seeds per pod, with a range from 2 to 12. However, in reciprocal crosses, 12 seeds per pod were detected, with a range of 4 to 17. Our findings were consistent with earlier research, indicating that seed-setting is more successful when the maternal parent has a greater ploidy level than the paternal parent [21,37,38,39].

Selfing progenies of the F_1_ hybrid (*B. rapa* ssp. (♀) × GM *B. napus* (♂)) and successive progenies of *B. rapa* ssp. *rapa* (KSF_2_ and KSF_3_) exhibited extremely low crossability index values and, thus, less compatibility. Although there is no experimental evidence to support this, we hypothesized that it was caused by pollen viability, pollen rejection, or pre-zygotic barriers during self-pollination. Thus, it may have an inhibition of pollen hydration and germination or pollen tube growth on the stigma [40,41,42]. Crossability is also influenced by reproductive barriers, which are dependent on parental fertility and pollen–pistil interactions [43,44]. Even if pollen germination and fertilization are successful, precocious or viviparous germination will occur, as previously reported [29,34,45]. Seed development is influenced by aberrant endosperm growth, embryo abortion, cross-species hybridization, parent ploidy levels, and hybridization directions [34,41]. The morphological characteristics and the number of progenies or individuals produced were strongly correlated with fitness [20,46,47,48]. In cluster analysis, 18 morphological characters were positively correlated with all the *B. rapa* ssp. and F_1_ hybrids, except the subspecies, ‘*pekinensis*’ and ‘*rapa*’ (Figure 1A). F_1_ selfing progenies of *B. rapa* ssp. *rapa* (♀) × GM *B. napus* (♂) had morphological characteristics similar to F_1_ hybrids (Figure 1B). However, they decreased their fitness values in all aspects compared to F_1_ hybrids. The transgene may have a direct contribution to their fitness increase/vigor or decrease/depression in the progenies [49,50]. In F_3_, the progenies belong to cluster 3, which is highly correlated with the number of seeds and number of pods (Figure 1B). Our results indicate that the generative characters in cluster 3 are similarly expressed in F_1_ hybrids, and F_1_ and F_3_ progenies (see Supplementary Tables S2 and S3). This information could be useful in the effective characterization of interspecific hybrid progenies of *B. rapa* (♀) × GM *B. napus* (♂) from self-pollination.

As expected, due to genetic imbalance, chromosome numbers may have varied significantly across all of these F_2_ and F_3_ hybrid selfing progenies. Interspecific hybridization causes chromosomal changes that can lead to transcriptional modifications that might affect the morphological characteristics of the plants [51]. Similarly, homologous recombination and increasing the chromosome numbers lead to reduced fitness [52], affect the seed yield [53], and induce genomic instability, thus reducing the probability of gene flow [54]. An assessment of relative fitness can prove the rational chromosome number variations in the interspecific hybrids. The interspecific hybridization and the chromosomal segregations were confirmed with Brassica A and C genome-specific SSR markers [26,55]. Through homologous recombination in F_1_ hybrids, they had a closer genetic similarity, a higher percentage of C genome, and transgene presence in all progenies than the backcross generation. These results concur with previously reported studies [6,56,57,58]. However, F_2_ and F_3_ progenies were found to have missed loci in both the A and C genomes. The homologous recombination between the A and C genomes leads to the deletion, rearrangements, and duplications of the chromosome (Zhang et al., 2016). Whereas nearly 46% of C genome loci were lost in KSBC_1_ progenies, only three A genome loci were lost. The transgene presence on one of the chromosomes of the C genome is transmitted at a low frequency. This suggests that the transgenes can more safely integrate into the C-chromosome than into the A chromosome [24]. That may be due to the higher level of homologous recombination with the AA-genome-containing maternal parent (*B. rapa* ssp. *rapa*). Based on the UPGMA cluster analysis results, KSF_2_ and KSF_3_ progenies were shown to be genetically distant from the KSBC_1_ generation. However, a few KSBC_1_ generations were more closely placed with KSF_2_ and KSF_3_ progenies.

## 4. Materials and Methods

### 4.1. Plant Material and Growth Conditions

Early flowering transgenic (GM) *Brassica napus* L.‘Youngsan’ (YS) (TG#39) (AACC, 2n = 38) was transformed with CAMV 35S-regulated bar and *BrAGL20* [59] and *B. rapa* L. ssp. *pekinensis* ‘Jangkang (JK) [29] and six subspecies of *B. rapa*: *B. rapa* L. ssp. *parachinensis* ‘Pakchoi (PC)’, *B. rapa* L. ssp. *chinensis* ‘Chaesim (CS)’, *B. rapa* L. ssp. *nipposinica* ‘Kyoungsoochae (KSC)’, *B. rapa* L. ssp. *narinosa* ‘Dachae (DC)’, *B. rapa* L. ssp. *Oleifera* ‘Soonmyouchae (SM)’, and *B. rapa* L. ssp. *rapa* ‘Kangwhasoonmu (KS)’ seeds were obtained from the National Agrobiodiversity Center, Jeonju, Republic of Korea. The seeds of GM *B. napus* were sown at three different times to ensure the synchronization of flowering time with different *B. rapa* ssp. All the plants were grown in individual container pots (21.5 cm) filled with a commercial horticultural soil mixture. Pots were spaced 10 cm apart and were watered every day until the flowers stopped blooming. The plants were maintained at an average day and night temperature of 25 ± 3 °C. All the experiments were conducted at the biosafety greenhouse at the National Institute of Agricultural Sciences, Jeonju, South Korea (Supplementary Figure S4).

### 4.2. Hybridization of GM B. napus with Different B. rapa ssp.

Interspecific hybridization experiments were performed by using *B. rapa* ssp. as a maternal parent (♀) and GM *B. napus* as a paternal parent (♂). In addition, we also perform hybridization with reciprocal combinations (Supplementary Figure S4). An average of 1328 young flower buds was used for artificial hand pollination in different plants for each crossing experiment. The emasculated *B. rapa* flower buds were pollinated with pollen from GM *B. napus* flowers the next day and then immediately covered with sealed, pre-labeled bags after pollination. Then, the plants were allowed to grow, and the fructification events of siliques were observed. We measured medium-sized pods (10 no.) for each plant to determine the crossability indexes for all of the cross-combination plants. *B. napus* and GM *B. napus* were used as standard controls, and the number of seeds per pod was calculated as a hybridization crossability index between GM *B. napus* and different *B. rapa* ssp. The resultant F_1_ hybrids were self-pollinated (5 plants), and produced F_2_ and F_3_ selfing progenies. Furthermore, the F_1_ hybrids (pollen donor) were crossed with *B. rapa* (seed parent), and produced BC_1_ progenies. For all of the progeny, the crossability index was calculated as the number of seeds obtained per pod. The survival rate (%) of seedlings after herbicide treatment was used to calculate the herbicide resistance rate. Briefly, seedlings were sprayed with 0.3% Basta (Bayer Crop Science GmbH, Manheim am Rhein, Germany) at the 4–5 leaf stage and again 4 days later, and seedling survival was measured at 4–7 days after the second application (details are in Supplementary Table S4). For the backcross generation detection of bar proteins in transgenic plants, a qualitative detection of bar proteins in the leaves of transgenic plants was conducted using a commercial immunostrip specific to bar proteins (Agrastrip^®^ seed & leaf TraitCheck LL, Company: Romer Labs) according to the manufacturer’s instructions (Supplementary Figure S5). PCR reactions for bar genes were performed according to Sohn et al. [29] (Supplementary Figures S6–S8).

### 4.3. Morphological Characteristics

The morphological characteristics (vegetative and generative) of all the parental lines and F_1_ hybrids, followed by the generation of 29 F_1_, 20 F_2_, and 23 F_3_ selfing progenies, were investigated (Supplementary Figures S9 and S10). The morphological characters of all the plant components were classified using the multigrade International Union for the Protection of New Varieties of Plants descriptors for *Brassica* [60]. The vegetative characters are as follows: PH, plant height; BS, branch segment; NOB, no. of branches (1,2,3). The generative characters are: NPF, no. of pollinated flowers; NOP, no. of pods; PSR, pod-setting ratio; NOS, no. of seeds; SPP, seeds per pod; VV, vivipary; NFS, non-filled seeds; FL, flower length; FW, flower width; FLD, flower diagonal; FIS, filament short; FIL, filament long; STL, style length (Supplementary Table S1).

### 4.4. Chromosome Numbers

The root tips were collected at 8 a.m. because of the high mitotic activity. Immediately after harvesting, the roots were pre-treated with 8-hydroxyquinoline at room temperature (RT) for 4 h. Following the pre-treatment, the root tips were rinsed with distilled water and treated with a 3:1 (*v*/*v*) mixture of ethanol and acetic acid. This was used to fix the pre-treated roots for 24 h at RT. The roots were rinsed again using distilled water and kept in 70% ethanol and stored at −20 °C until the roots were ready to be used. The fixed roots were washed with distilled water and the meristematic portions were cut off. The cells were then immersed in a hydrolyzed enzyme buffer (Cytohelicase 250 mg, Cellulose 250 mg, Pectolyase 250 mg in 25 mL of 0.01 M citrate) for 1 h at 37 °C. After washing the enzyme, the roots were gently tapped or crushed with a pin. Then, a drop of acetic acid (60%) was added to clean and evenly distribute the roots, and they were placed in an oven at 46 °C for 2 min. Finally, the slides were counterstained with Vectashield (H-1000) with DAPI (4,6-diamidino-2-phenylindole, Sigma), and covered with filter paper by applying firm thumb pressure. To avoid autofluorescence, the prepared slides were treated with a drop of immersion oil before being examined under a Nikon Eclipse 50i fluorescence microscope at a magnification of 100×. The method described here is a slight modification to the protocols of Tagashira et al. [61] and Hoshi [62].

### 4.5. SSR Analysis

The SSR markers used in this study were derived from a previous report by Zhang et al., 2016, and the markers which can produce two bands were selected based on a comparison between the A and C genomes in *B. napus* (Supplementary Table S5). Among them, 17 SSR primers were generated, with clearly distinguishable bands, which were used for further analysis. The genomic DNA was extracted from leaf tissue using the cetyl trimethyl ammonium bromide (CTAB) method [63]. The polymerase chain reaction (PCR) mixture in a 20 µL volume contains forward and reverse primer (1 µL) (10 picomol each), gDNA (1 µL), Taq PCR mix (http://cells-safe.com/, accessed on 14 February 2022), and RNAase-free water (18 µL). The PCR amplification was performed in a thermal cycler (Biometra Thermal cycler) with the following conditions: an initial denaturing step at 95 °C for 3 min; followed by 35 cycles of 95 °C for 30 s, 56 °C for 30 s, 72 °C for 30 min, and 72 °C for 10 min [55]. The amplified PCR products were visualized using a QSEP400 high-throughput gel electrophoresis system (Qsep400 multi-channel Bio-fragment analyzer). The amplifications were scored on the basis of the presence or absence of bands (H: 1,1; A: 1,0; C: 0,1;) and were depicted as binary characters. To find the genetic relationships among the progenies, Jaccard’s distance matrix was plotted using DARwin software for Windows version (6.0.021) [64], and clustering was carried out using the unweighted pair group method and arithmetic average (UPGMA). The resulting phylogenetic tree was exported using Evolview [65] for graphical annotation.

### 4.6. Statistical Analysis

The data on morphological characteristics were analyzed using the R program v 4.1.2 (https://cran.r-project.org/bin/windows/base/old/4.1.2/R-4.1.2-win.exe, accessed on 6 September 2022). Using the package, ‘agricolae’ [66], a one-way analysis of variance (ANOVA), followed by Tukey’s mean separation, was carried out with a significance difference at *p* = 0.05. Principal component analysis, followed by a hierarchical clustering analysis, was performed to assess the relationship among the genotypes based on morphological characteristics using FactoMinerR [67] and Factoextra [68]. Prior to the multivariate analyses, missing data were imputed with the missMDA [69] package.

## 5. Conclusions

The GM *B. napus* can effectively hybridize with different subspecies of *B. rapa* through artificial hand pollination in a controlled environment. In particular, it can produce several viable and fertile generations (F_1_, F_2_, F_3_, and BC_1_) with *B. rapa* ssp. *rapa*, and can transfer the herbicide-resistant transgene to their progenies. In greenhouse conditions, artificial hand pollination with transgenic *B. napus* resulted in a 100% outcrossing rate. However, in field conditions, spontaneous hybridization has an outcrossing rate, ranging from 0.02 to 2.78% in field conditions [34,70]. Due to several environmental factors, the outcrossing rate is much lower compared to greenhouse conditions. According to our data, greenhouse containment is the most successful approach for preventing natural gene flow. So far, no examples of greenhouse containment failure have been observed. The few conditions that have a significant impact on the outcrossing rate are unlikely to occur naturally: (i) in nature, there will be fewer flowering possibilities for transgenic *B. napus* and *B. rapa* at the same period; (ii) the *B. rapa* flowering period was controlled using the vernalization process; (iii) the young flower buds are the determining factor for successful cross-pollination/hybridization in other subspecies; and (iv) the pollen of transgenic *B. napus* was manually transferred by artificial hand pollination to *B. rapa* ssp., and the plants were maintained at controlled conditions throughout their life cycle. It is necessary to understand the transgene expression characteristics of hybrid progenies to assess the transgene persistence. Further gene flow studies are needed for the enhanced understanding of the process, and to assess its impacts on the environment and ecology.

## Figures and Tables

**Table 1:** Details of cross-compatibility with *B. rapa* ssp. and GM *B. napus*.

Crossing Materials	Cross-Combination	No. of Pollinated Flowers	No. of Pods	Pod-Setting Ratio (%)	Total No. of Seeds	Vivipary (%)	Empty Seeds (%)	Crossability Index Avg. of 10 Pods/Plant (No. of Seeds/Pods)
Parental	*B. napus*	410	282	68.8	6073	89 (1.47)	249 (4)	21.5 ± 0.7 ^b^
	GM *B. napus*	1197	786	65.6	4772	53	123	10.1 ± 5.3 ^e^
	*B. rapa* ssp. *pekinensis*	268	159	59.4	1657	12 (0.72)	454 (27)	10.6 ± 0.7 ^c^
	*B. rapa* ssp. *parachinensis*	1741	641	36.8	4153	174 (4.2)	610 (14.7)	13.7 ± 1.2 ^d^
	*B. rapa* ssp. *chinensis*	792	292	36.9	3723	161 (4.3)	544 (14.6)	25.1 ± 2.3 ^a^
	*B. rapa* ssp. *nipposinica*	1535	421	27.4	2704	61 (2.3)	873 (32.3)	13.3 ± 1.1 ^c^
	*B. rapa* ssp. *narinosa*	1211	656	54.2	5338	54 (1.0)	2058 (38.6)	19.8 ± 1.5 ^b^
	*B. rapa* ssp. *oleifera*	1875	603	32.2	3799	158 (4.2)	827 (21.8)	15.0 ± 1.8 ^e^
	*B. rapa* ssp. *rapa*	285	201	70.5	147	1 (0.7)	14 (9.5)	6.1 ± 1.2 ^c^
F_1_ Hybrids	*B. rapa* ssp. *pekinensis ♀* × GM *B. napus♂*	1282	540	42.1	1926	518 (26.9)	403 (20.9)	15.4 ± 1.7 ^ab^
	*B. rapa* ssp. *parachinensis♀* × GM *B. napus ♂*	220	155	70.5	1932	1138 (58.9)	144 (7.5)	15.4 ± 2.7 ^ab^
	*B. rapa* ssp. *chinensis♀* × GM *B. napus ♂*	390	217	55.6	1758	1250 (71.1)	385 (21.9)	13.6 ± 1.5 ^abc^
	*B. rapa* ssp. *nipposinica♀* × GM *B. napus ♂*	417	157	37.6	1054	769 (73)	224 (21.3)	12.3 ± 1.4 ^bc^
	*B. rapa* ssp. *narinosa♀* × GM *B. napus ♂*	616	421	68.3	3718	1415 (38.1)	734 (19.7)	16.9 ± 2.6 ^a^
	*B. rapa* ssp. *oleifera ♀* *×* GM *B. napus*	703	411	58.5	3936	1145 (29.1)	2498 (63.5)	16.2 ± 2.8 ^a^
	*B. rapa* ssp. *rapa* *♀* × GM *B. napus* ♂	580	449	77.4	748	170 (22.7)	370 (49.5)	11.0 ± 2.8 ^c^
Reciprocal Combinations	GM *B. napus ♀* × *B. rapa* ssp. *pekinensis ♂*	843	380	45.1	2595	25 (1.0)	75 (2.9)	14.2 ± 1.1 ^bc^
	GM *B. napus ♀* × *B. rapa* ssp. *parachinensis* *♂*	164	95	57.9	1522	21 (1.4)	49 (3.2)	24.4 ± 2.7 ^d^
	GM *B. napus ♀* × *B. rapa* ssp. *chinensis* *♂*	105	71	67.6	1112	1 (0.1)	17 (1.5)	22.1 ± 1.7 ^a^
	GM *B. napus ♀* × *B. rapa* ssp. *nipposinica* *♂*	175	107	61.1	1864	79 (4.2)	81 (4.3)	27.5 ± 2.9 ^c^
	GM *B. napus ♀* × *B. rapa* ssp. *narinosa* *♂*	169	119	70.4	1464	25 (1.7)	42 (2.9)	19.2 ± 1.6 ^b^
	GM *B. napus ♀* *×* *B. rapa* ssp. *oleifera* *♂*	167	117	70.1	1649	53 (3.2)	36 (2.2)	20.8 ± 2.4 ^d^
	GM *B. napus ♀* × *B. rapa* ssp. *rapa* *♂*	913	299	32.7	1315	33 (2.5)	74 (5.6)	11.9 ± 1.5 ^c^
F_1_ Hybrid (Selfing)	*B. rapa* ssp. *pekinensis♀* *×* GM *B. napus ♂*	4053	-	-	-	-	-	-
	*B. rapa* ssp. *parachinensis* ♀ × GM *B. napus* ♂	1245	5	0.4	4	1 (25)	-	0.8 ± 0.8 ^ab^
	*B. rapa* ssp. *chinensis* ♀ × GM *B. napus* ♂	1613	109	6.8	165	17 (10.3)	51 (30.9)	1.2 ± 0.4 ^a^
	*B. rapa* ssp. *nipposinica* ♀ × GM *B. napus* ♂	1415	4	0.3	3	-	2 (66.7)	0.75 ± 0.5 ^a^
	*B. rapa* ssp. *narinosa* ♀ × GM *B. napus* ♂	2735	23	16.3	25	1 (4)	9 (36)	0.85 ± 0.6 ^a^
	*B. rapa* ssp. *oleifera* ♀ × GM *B. napus* ♂	1683	200	11.9	240	17 (7.1)	147 (61.3)	1.4 ± 0.5 ^b^
	*B. rapa* ssp. *rapa* ♀ × GM *B. napus* ♂	6382	551	8.63	877	61 (7)	248 (28.3)	1.6 ± 0.7 ^ab^

GM *B. napus* (TG#39), *B. rapa* ssp: *pekinensis* ‘Jangang’ (JK); *parachinensis* ‘Pakchoi’ (PC); *chinensis* ‘*Chaesim*’ (CS); *nipposinica* ‘Kyoungsoochae’ (KSC); *narinosa* ‘Dachae’ (DC); *Oleifera* ‘Soonmuyouchae’ (SM); *rapa* ‘Kangwhasoonmu’ (KS). ^a–e^ indicate statistical significance between the different crossing materials. One-way ANOVA performed separately for each group of crossing material and followed by Tukey’s HSD test at *p* < 0.05.

## Data Availability

Not applicable.

## Supplementary Materials

The following supporting information can be downloaded at: https://www.mdpi.com/article/10.3390/ijms231810512/s1.
