# Supplementary material for: Characteristics and Fitness Analysis through Interspecific Hybrid Progenies of Transgenic Brassica napus and B. rapa L. ssp
Source: Int J Mol Sci. 2022 Sep 10;23(18):10512. doi: 10.3390/ijms231810512 (PMC9506035; doi:10.3390/ijms231810512)
Supplement: Supplementary file 1 [file ijms-23-10512-s001.zip › ijms-1875774-supplementary/Supplementary Figures_Sohn et al. 2022.pdf]

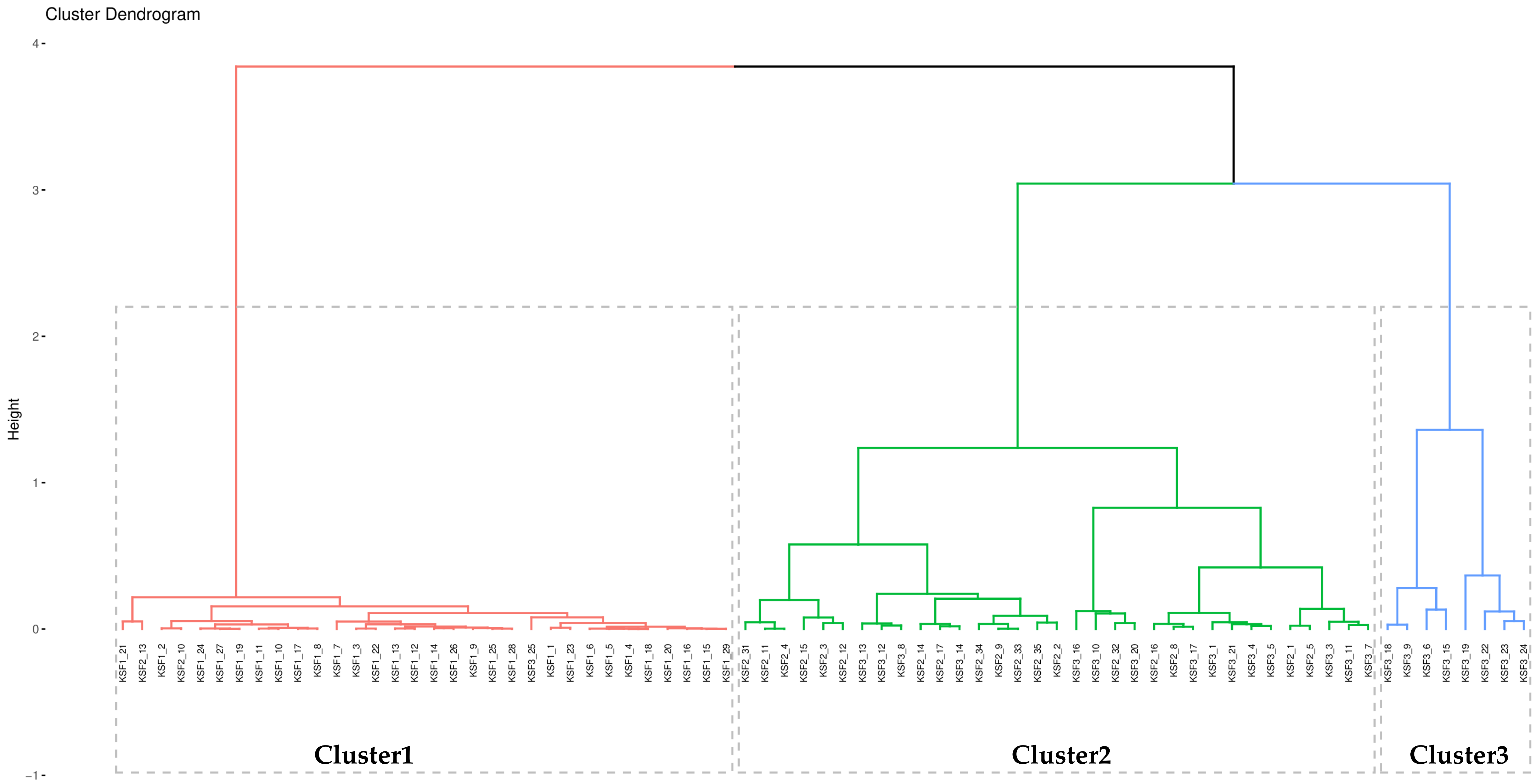

Figure S1. Cluster representation of selfing progenies of *B. rapa* ssp. *rapa* x GM *B. napus* with 18 agricultural characteristics. Details provided in the **Supplementary Table S3.**

	C01b	C02a2	C02d2	C03a2	C03b	C04b2	C04c	C04d	C06b2	C06d2	C07c2	C07d2	C08a2	C08b2	C08c2	C08d	C09c2
YS	H	H	H	H	H	H	H	H	H	H	H	H	H	H	H	H	H
KS	A	A	A	A	A	A	A	A	A	A	A	A	A	A	A	A	A
KSF1	H	H	H	H	H	H	H	H	H	H	H	H	H	H	H	H	H
Cabbage	C	C	C	C	C	C	C	C	C	C	C	C	C	C	C	C	C
Broccoli	C	C	C	C	C	C	C	C	C	C	C	C	C	C	C	C	C
1KSBC1	H	A	A	H	H	A	H	A	H	H	H	H	A	H	H	H	A
2KSBC1	H	H	H	H	H	A	A	A	H	H	A	A	A	A	A	A	A
3KSBC1	H	A	A	H	H	A	H	A	H	A	H	H	A	H	H	H	H
4KSBC1	A	H	H	H	H	H	H	H	H	H	H	H	A	H	H	H	A
5KSBC1	A	A	H	A	A	A	A	A	A	A	A	A	A	A	A	A	A
6KSBC1	H	H	A	A	A	H	H	H	A	H	A	A	A	H	H	A	H
7KSBC1	H	A	A	H	H	H	H	H	H	H	A	A	A	A	A	A	A
8KSBC1	H	A	H	H	H	H	H	H	H	H	A	A	A	H	H	H	A
9KSBC1	A	H	A	A	A	A	A	A	A	A	H	H	A	A	A	A	A
10KSBC1	H	A	H	H	H	H	H	H	H	A	H	H	A	H	H	H	H
11KSBC1	H	A	H	H	H	H	H	H	H	A	H	H	A	H	H	H	A
12KSBC1	A	H	A	A	H	A	A	A	H	H	A	A	A	H	H	H	A
13KSBC1	A	H	A	A	A	A	H	A	A	A	A	A	A	H	H	H	A
14KSBC1	A	A	H	H	H	A	H	A	H	A	H	H	A	A	A	A	H
15KSBC1	H	A	A	H	H	H	H	H	H	H	A	A	A	H	H	H	H
16KSBC1	H	H	H	H	H	H	H	H	H	H	H	H	A	H	H	H	A
17KSBC1	H	A	H	H	H	A	A	A	H	A	A	A	H	H	H	H	A
18KSBC1	H	H	A	H	H	H	H	H	H	A	H	H	H	H	H	H	H
19KSBC1	H	H	H	H	H	H	H	H	H	H	H	H	H	H	H	H	A
20KSBC1	H	A	H	A	A	A	H	A	A	A	A	A	A	A	A	A	A
21KSBC1	H	A	A	A	A	A	A	A	A	H	A	A	A	A	H	A	A
22KSBC1	A	H	H	H	H	A	H	A	H	H	H	H	H	H	H	A	A
23KSBC1	A	A	H	H	H	A	H	A	H	H	A	A	H	H	H	A	A
24KSBC1	H	A	A	A	A	A	A	A	A	A	A	A	A	A	A	A	A
25KSBC1	A	A	H	H	H	A	H	A	H	A	H	H	A	A	A	A	A
26KSBC1	H	A	H	H	H	H	H	H	H	A	H	H	A	A	A	A	H
27KSBC1	H	A	A	H	H	H	H	H	H	H	H	H	H	H	H	H	A
28KSBC1	H	A	A	A	A	A	H	A	A	A	A	A	A	A	A	A	A
29KSBC1	H	A	H	H	H	A	H	A	H	H	H	H	H	H	H	H	A
30KSBC1	H	A	A	H	H	H	H	H	H	H	A	A	A	A	A	A	A
31KSBC1	H	A	H	A	A	H	H	H	A	A	A	A	A	A	A	A	H
32KSBC1	H	H	H	H	H	A	H	A	H	A	A	A	H	H	H	H	A
33KSBC1	A	H	H	A	A	H	H	H	A	H	H	H	A	H	H	A	H

Figure S2. Chromosome wise assessed the frequencies of interspecific hybrids *B. rapa* ssp. *rapa* (♀) x GM *B. napus* (♂) (KS) of BC_1 generation were indicated by SSR markers.

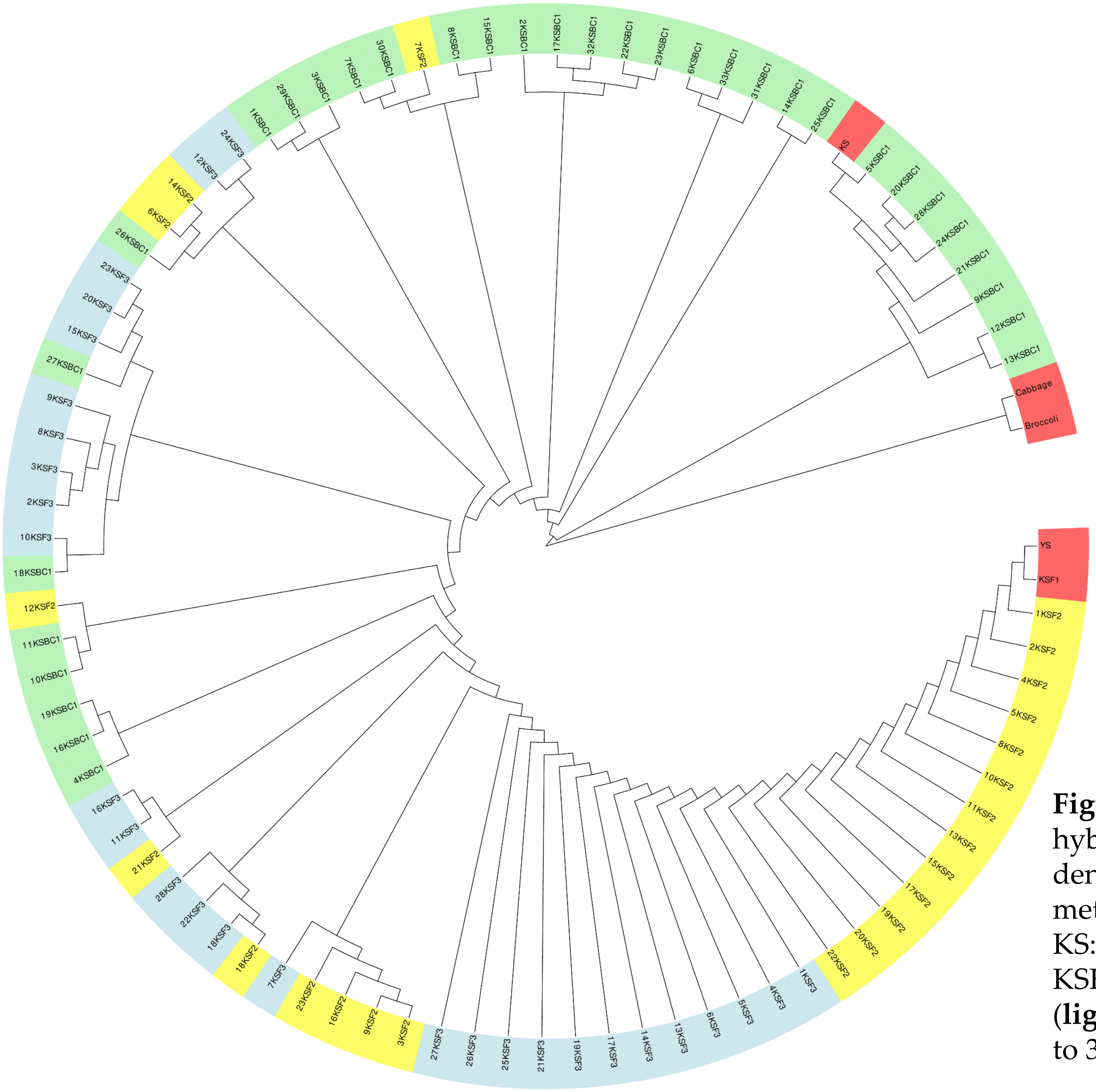

Figure S3. Genetic distance matrix between KSF_2, KSF_3 hybrids and backcross generation ($KSBC_1$). The dendrogram was designed using the UPGMA clustering method based on Jaccard's distance matrix. YS: *B. napus*; KS: *B. rapa* ssp. *rapa*, *B. oleracea* var. (Cabbage, Broccoli), KSF_1 (**Red**); $1KSF_2$ to $23KSF_2$ (**yellow**): $1KSF_3$ to $28KSF_3$ (**lightblue**) F2 and F3 hybrids selfing progenies; $1KSBC_1$ to $33KSBC_1$: backcross generation (**green**).

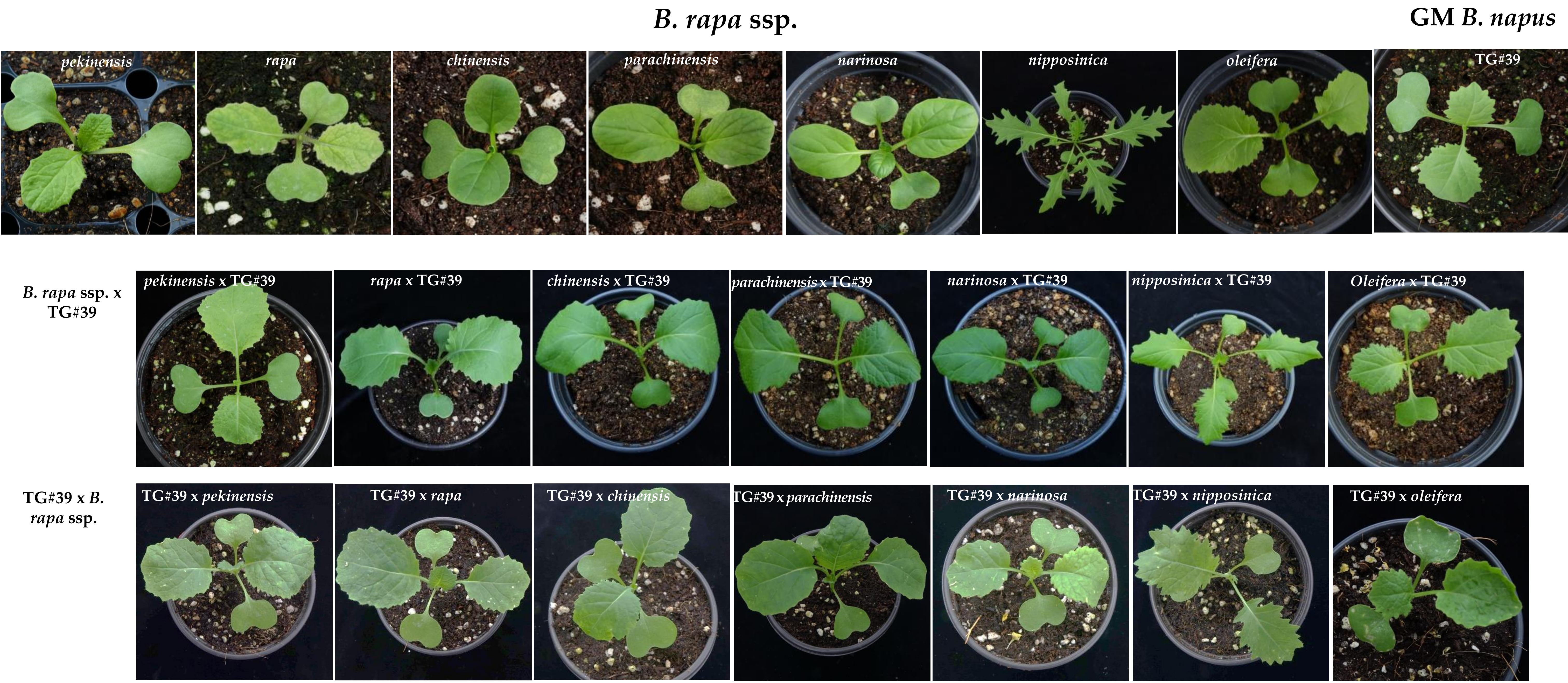

Figure S4. A morphological representation of non GM B. napus (YS), *B. rapa* ssp. and GM *B. napus* (TG#39). Parental cross combination of *B. rapa* ssp (♀) x GM *B. napus* (♂) and reciprocal combination of GM *B. napus* (♂) x *B. rapa* ssp (♀).

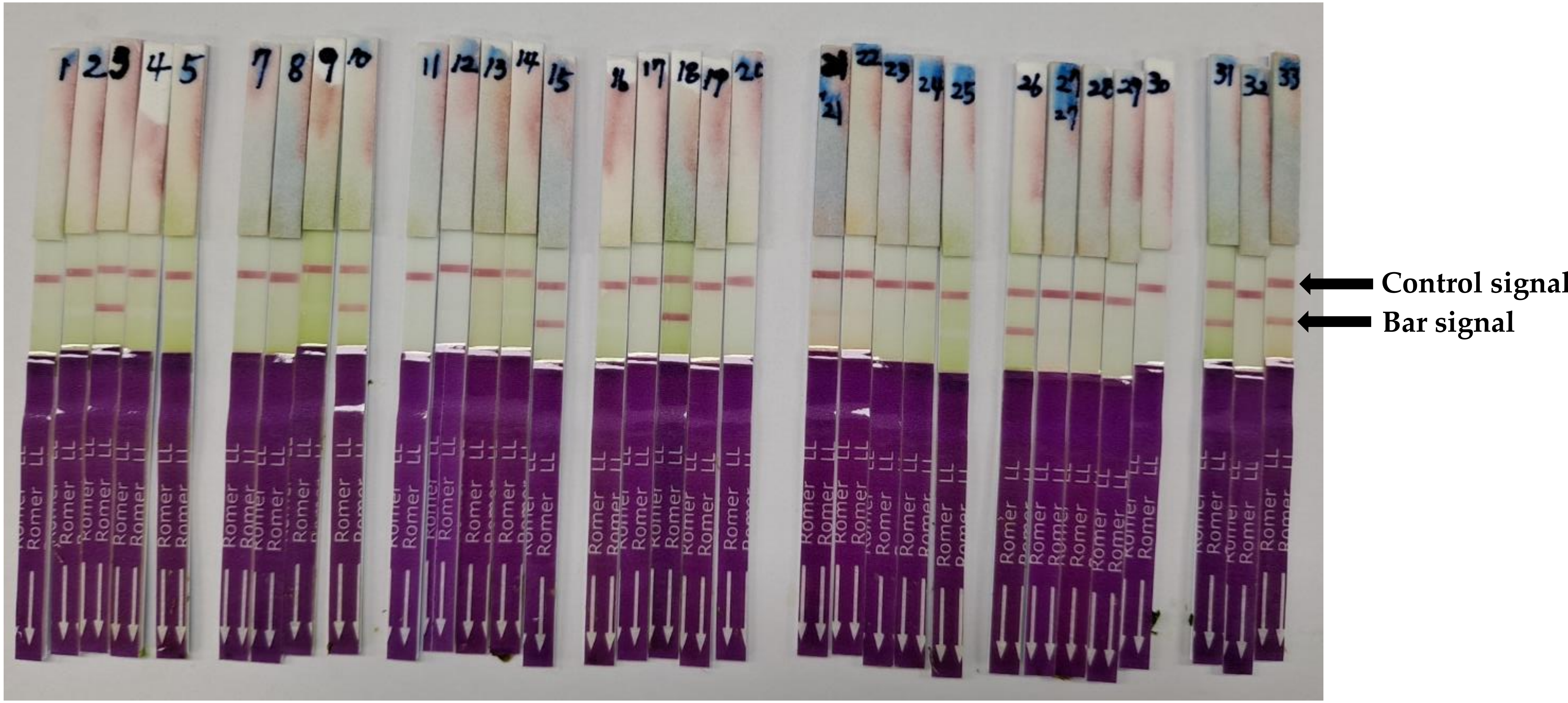

Figure S5. Immunostrip specific to bar protein detection results for *B. rapa* ssp. rapa (KS) 1–33 backcross generations.

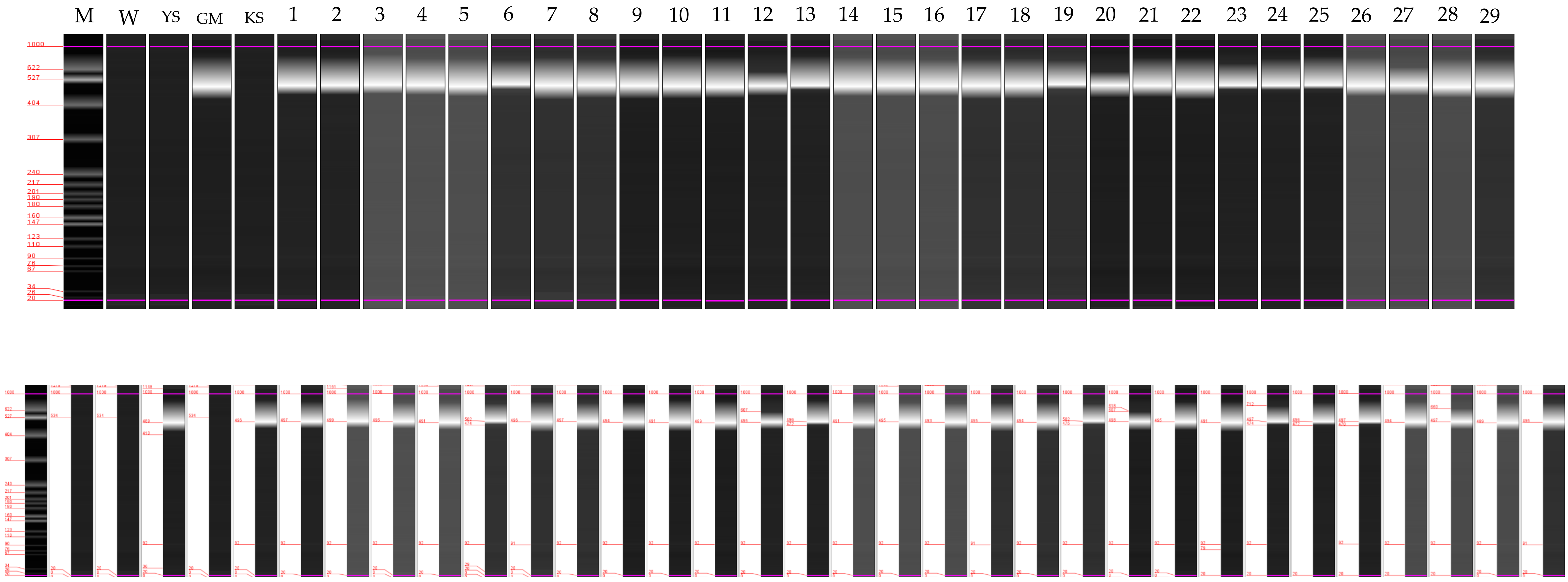

Figure S6. Polymerase chain reaction for detection of transgene (*bar* gene) in F1 hybrids *B. rapa* ssp. *rapa* (KS).
M – Marker; W – Negative control (Water); YS – *B. napus;* GM – GM *B. napus;* KS – *B. rapa* ssp. *rapa;* 1–29 – F1 progenies.

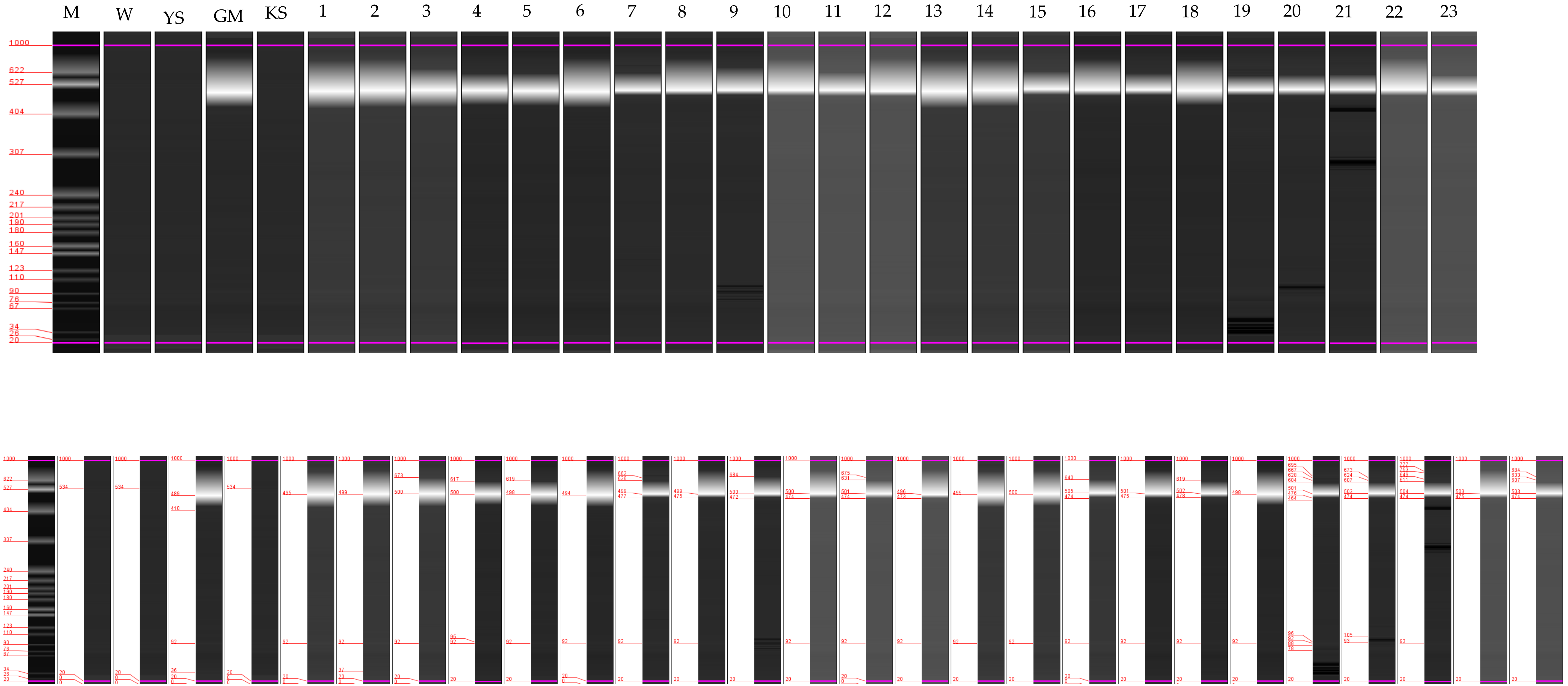

Figure S7. Polymerase chain reaction for detection of transgene (*bar* gene) in F2 hybrids *B. rapa* ssp. *rapa* (KS). M – Marker; W – Negative control (Water); YS – *B. napus;* GM – GM *B. napus;* KS – *B. rapa* ssp. *rapa;* 1–23 – F2 progenies.

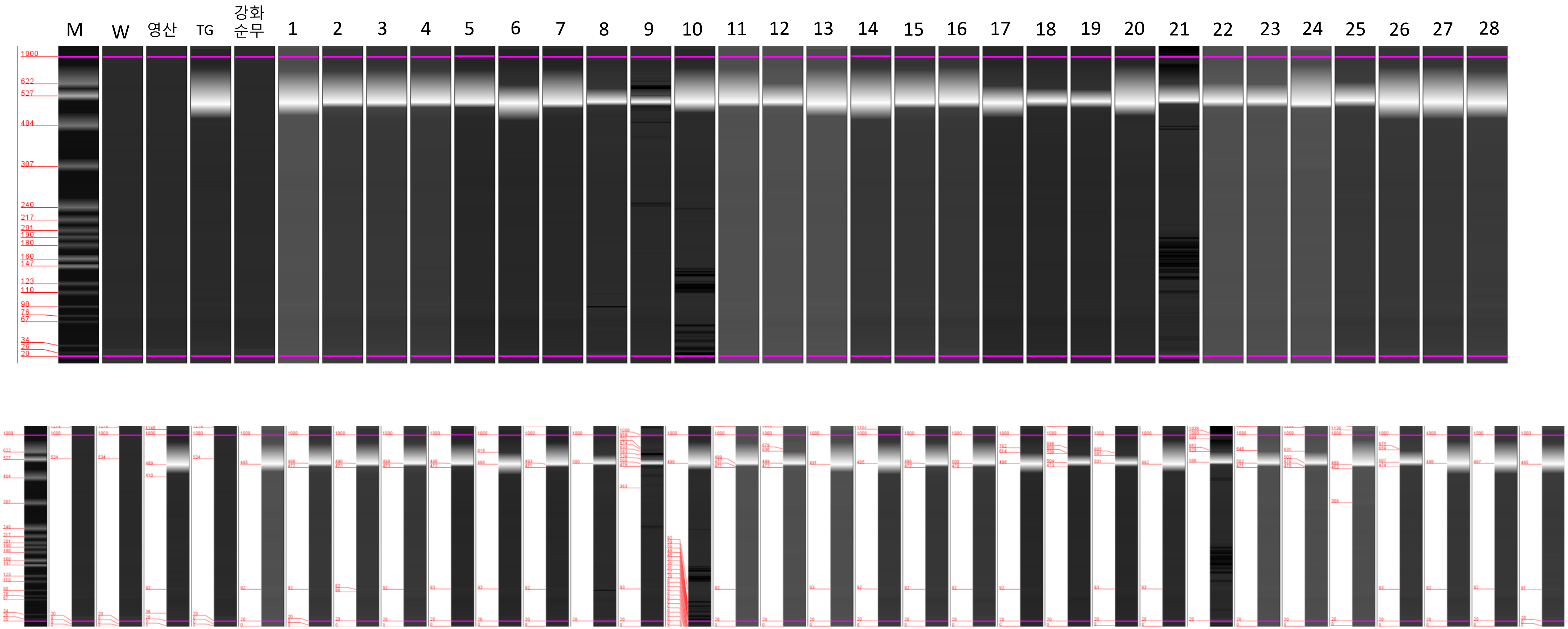

Figure S8. Polymerase chain reaction for detection of transgene (*bar* gene) in F3 hybrids *B. rapa* ssp. *rapa* (KS).
M – Marker; W – Negative control (Water); YS – *B. napus;* GM – GM *B. napus;* KS – *B. rapa* ssp. *rapa;* 1–28 – F3 progenies.

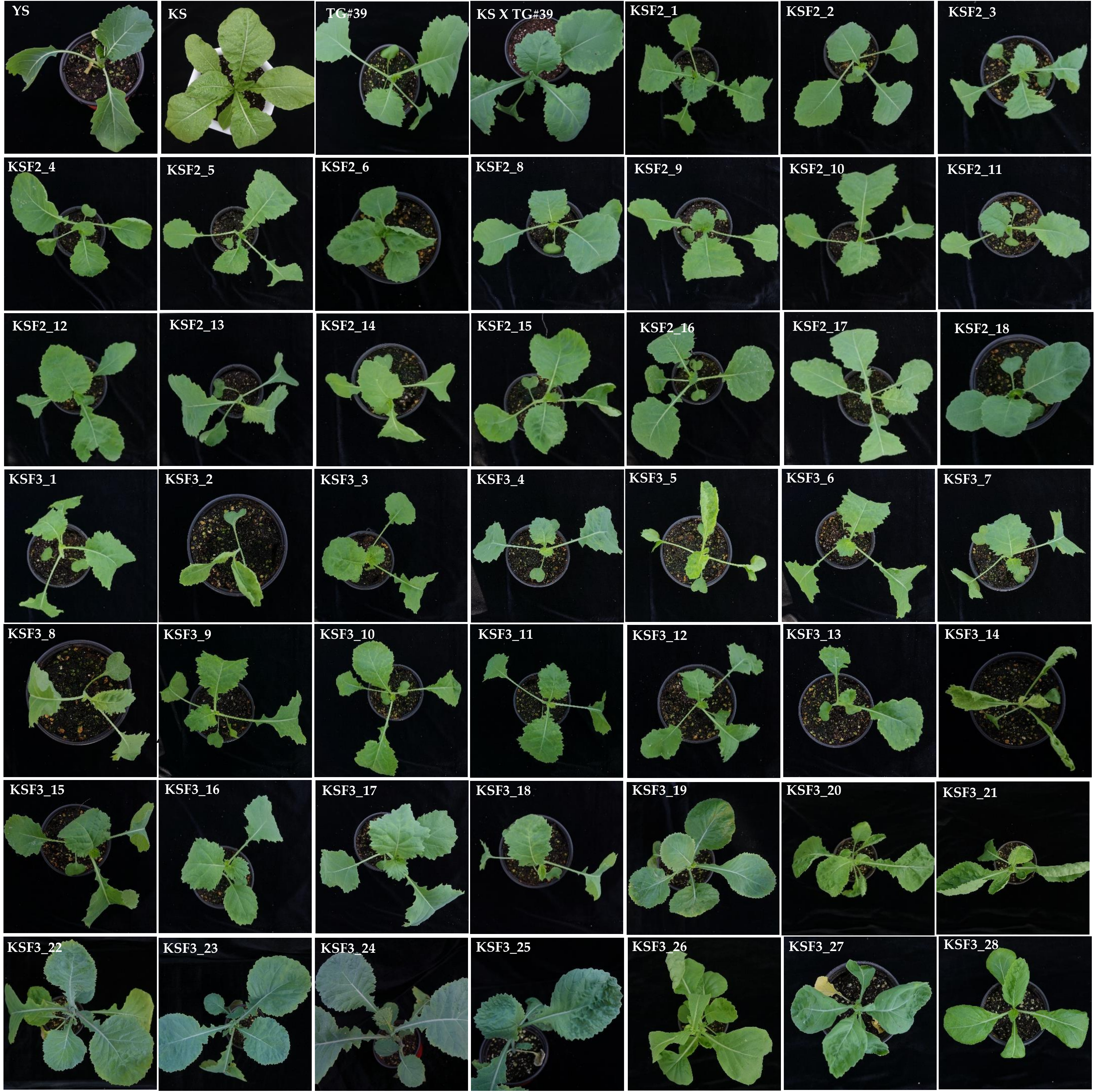

Figure S9. A morphological representation of non GM *B. napus* (YS), *B. rapa* ssp. and GM *B. napus* (TG#39). Cross combination of *B. rapa* ssp. *rapa* (♀) x GM *B. napus* (♂) F_1 hybrids and selfing generation of KSF_2 and KSF_3.

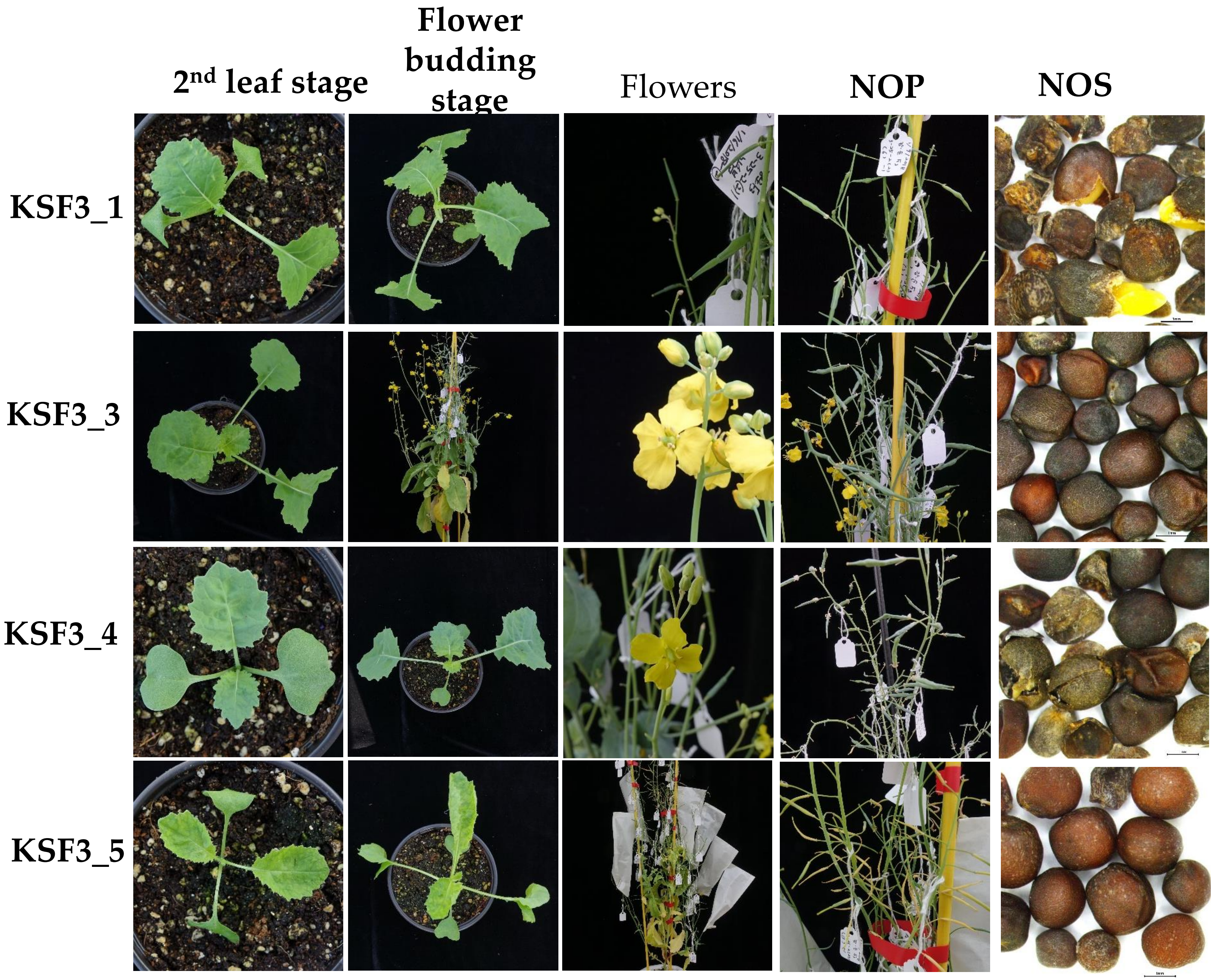

Conti.,

Figure S10. A morphological representation cross combination of *B. rapa* ssp. *rapa* (♀) x GM *B. napus* (♂) selfing generation of KSF_3

Conti.,

KSF3_6

KSF3_7

KSF3_9

KSF3_10

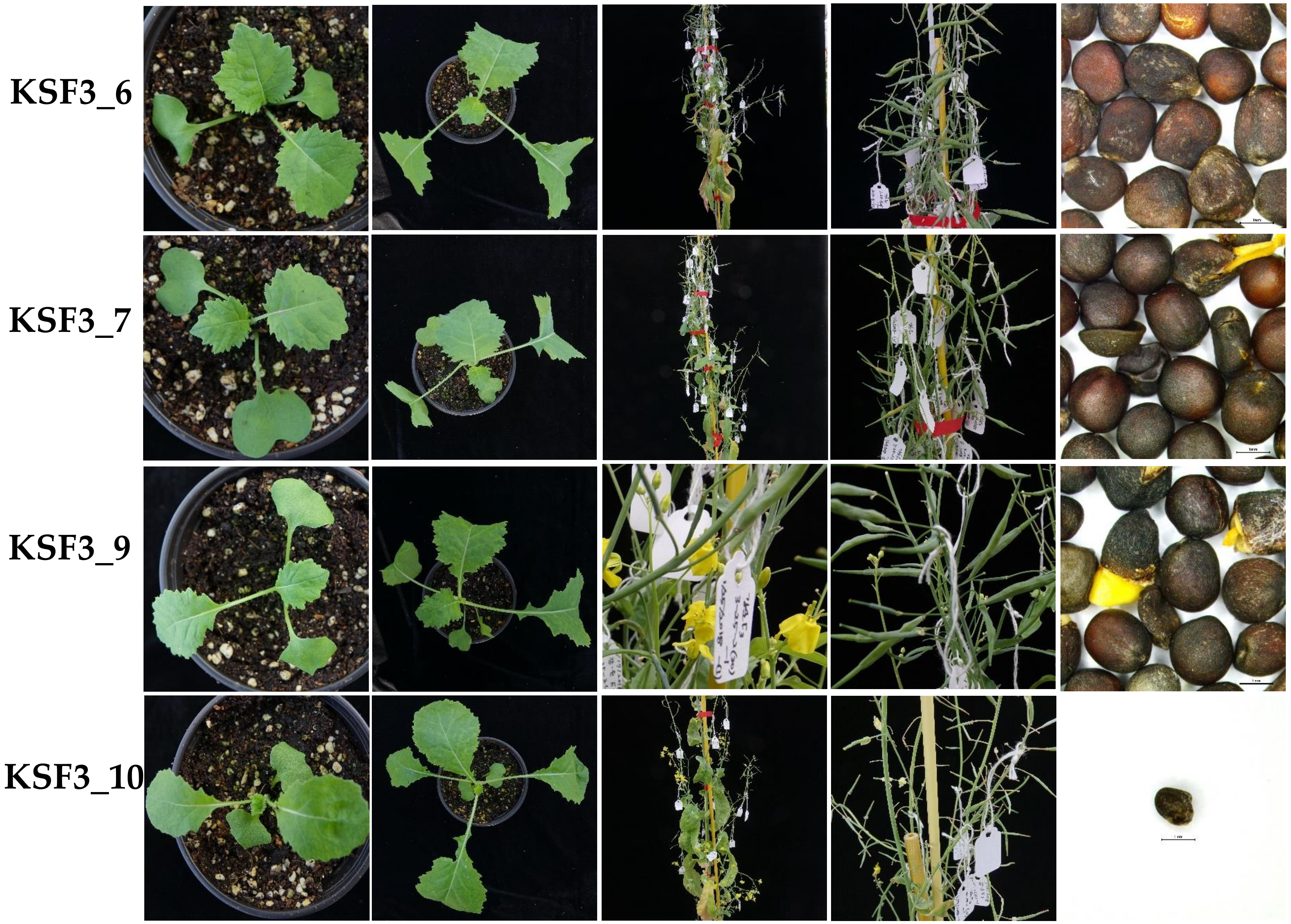

Figure S10. A morphological representation cross combination of *B. rapa* ssp. *rapa* (♀) x GM *B. napus* (♂) selfing generation of KSF_3

Conti.,

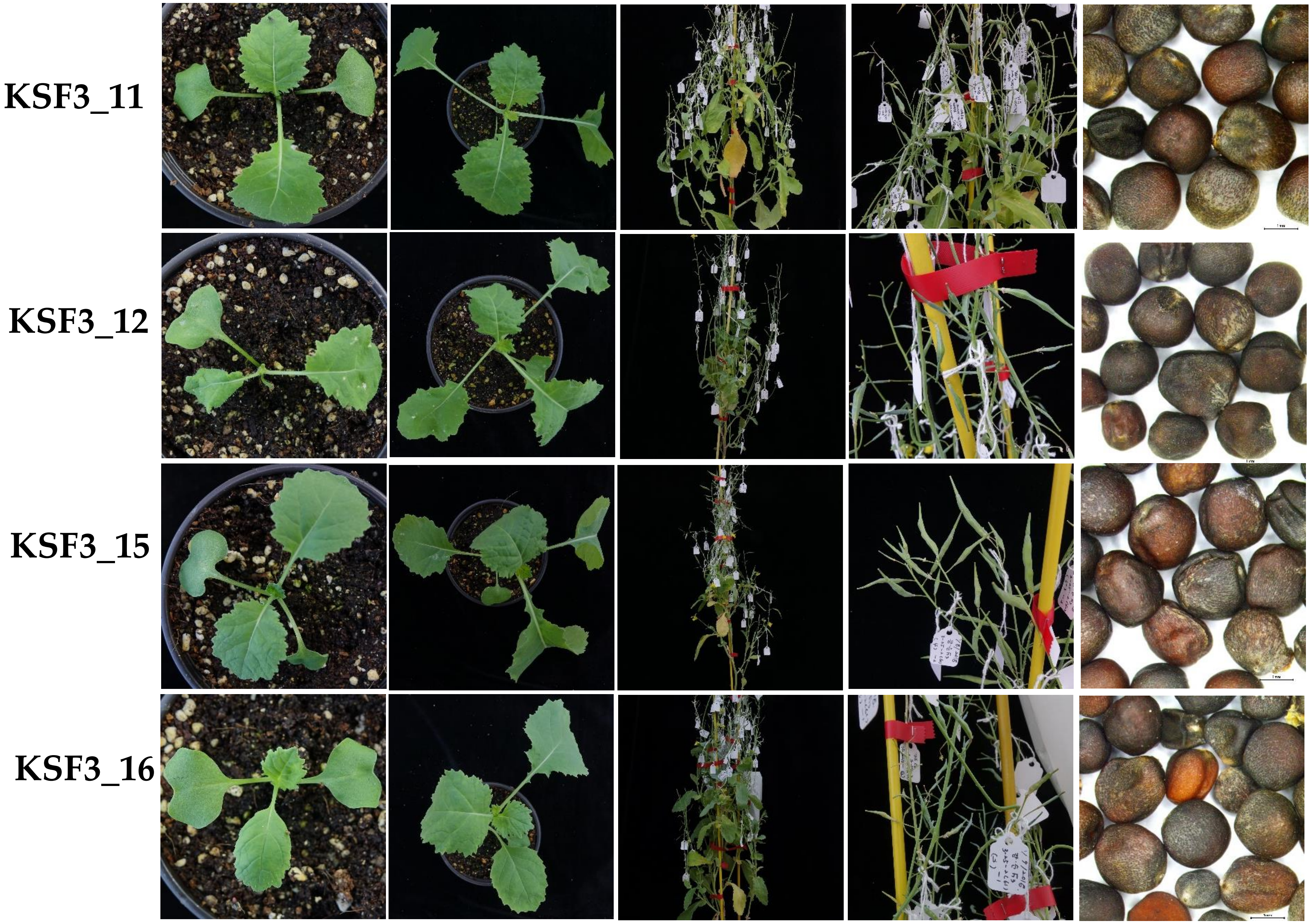

Figure S10. A morphological representation cross combination of *B. rapa* ssp. *rapa* (♀) x GM *B. napus* (♂) selfing generation of KSF_3

Conti.,

KSF3_19

KSF3_20

KSF3_21

KSF3_22

KSF3_23

KSF3_24

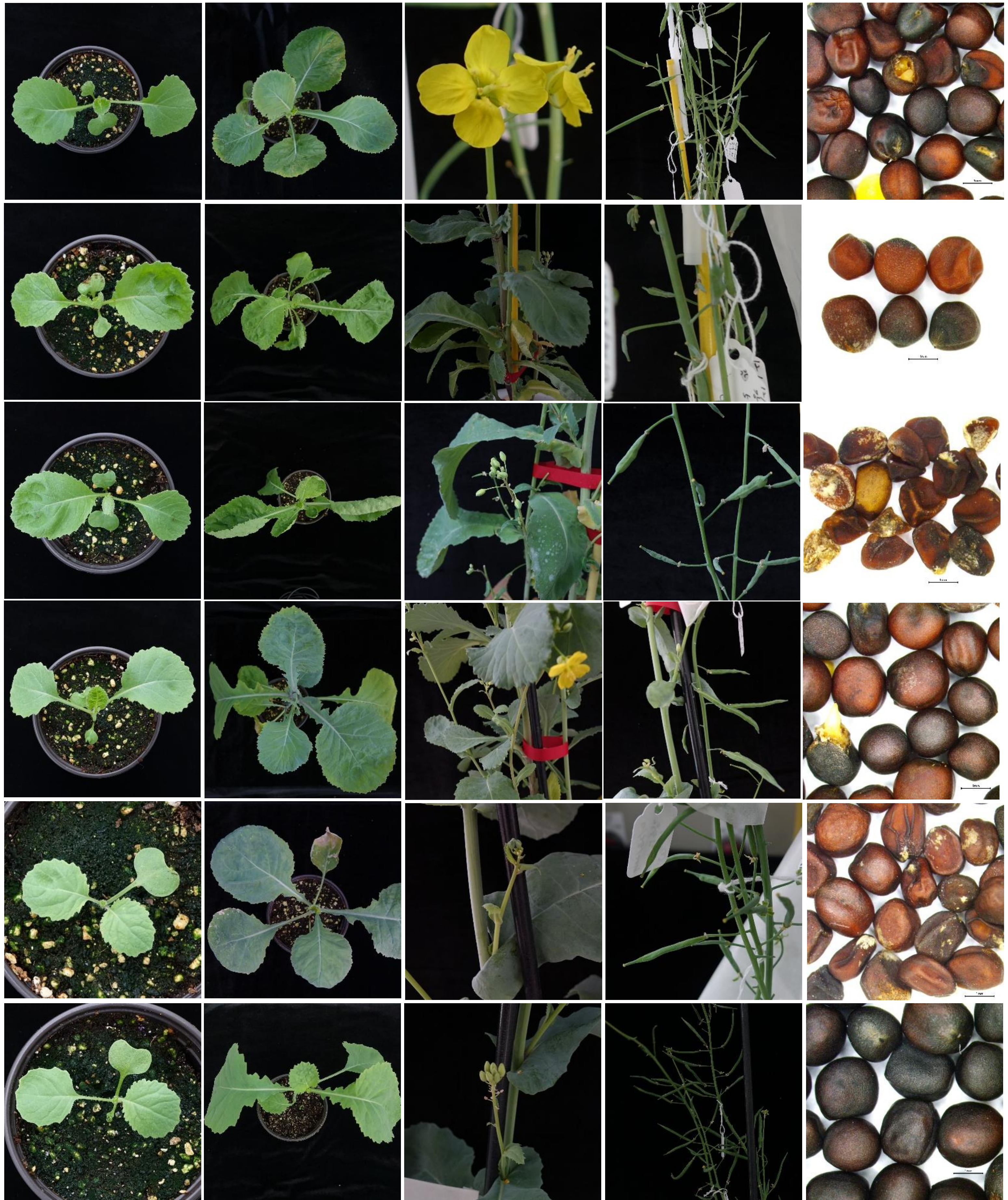

Figure S10. A morphological representation cross combination of *B. rapa* ssp. *rapa* (♀) x GM *B. napus* (♂) selfing generation of KSF_3
